# First Reported Use of Diluted HArmonyCa™ for Neck Rejuvenation: A Report of Two Cases

**DOI:** 10.7759/cureus.82181

**Published:** 2025-04-13

**Authors:** Apul Parikh

**Affiliations:** 1 Plastic Surgery, Dr Apul Parikh Clinic, London, GBR

**Keywords:** calcium hydroxyapatite, gais, harmonyca™, neck rejuvenation, transverse neck lines

## Abstract

Neck rejuvenation is one of the most commonly sought-after treatments in aesthetic medicine. This study demonstrates neck rejuvenation using dilute HArmonyCa™. To date, there have been no publications regarding the efficacy of HArmonyCa™ in the neck. Two female patients who presented for neck rejuvenation were injected using dilute HArmonyCa™. The product was diluted using a ratio of 1:1, using normal saline. The patients and the injecting doctor completed the Global Aesthetic Improvement Scale (GAIS) to assess the results. The Allergan Transverse Neck Lines (ATNL) scale was also used to rate the severity of neck lines, and a one-point improvement was used as a marker of a successful treatment. The mean age of the patients was 55 years. Both patients achieved a one-point improvement in the GAIS score. Prior to treatment, Patient 1 was rated “moderate” (2 out of 4) on the ATNL. Patient 2 was rated “severe” (4 out of 4). Post-treatment, both patients dropped down to 1. No adverse events were noted. Dilute HArmonyCa™ was well tolerated in the neck. The patients achieved improvement in their neck lines, with a one-point improvement using validated scales. This case series paves the way for a larger study, with a longer follow-up.

## Introduction

Ageing in the neck is a multifactorial process influenced by intrinsic and extrinsic factors, and it is often one of the earliest visible signs of ageing [1]. The neck's ageing process often remains a challenge for both patients and clinicians due to its complex anatomy and continuous exposure to environmental factors. There is thinning of the skin as well as a decrease in the amount of collagen and elastin [2]. Subsequently, there is sagging of the neck, dryness of the soft tissue, flaccidity, and the development of transverse neck lines. Seeking rejuvenation for ageing necks is one of the most common reasons that patients attend nonsurgical aesthetic clinics.

HArmonyCa™ (Allergan Aesthetics) is a dermal injectable product designed for treatment in the face. It consists of calcium hydroxyapatite (CaHA) spheres suspended in a cross-linked sodium hyaluronate (HA) gel [3]. The HA element may provide a mild immediate lifting effect. The CaHA microspheres work within the dermis to form a scaffold that supports the internal growth of fibroblasts, which then induce the formation of collagen fibres. This case series explores its off-label use in neck rejuvenation.

## Case presentation

Methods

Two females attended the author's clinic, seeking neck rejuvenation. They had no significant medical history and had not undergone any previous treatments to the neck.

Technique

HArmonyCa™ is a highly cohesive product and has a high elasticity [3]. The treatments were thus performed using a 22-gauge cannula. As the neck skin is particularly thin, the product was diluted using 1 ml of normal saline in an aseptic manner. This helped ensure that there were no lumps in the product. The neck region was further massaged manually using damp gauze post-treatment to ensure smooth and equal distribution of the product in the region.

Outcome measures

Prior to treatment, the patients were asked to evaluate themselves using the Global Aesthetic Improvement Scale (GAIS), which measures satisfaction with aesthetic treatments (Table 1). The same self-assessment was repeated three months post-treatment. In parallel, the authors independently assessed and scored the patients’ outcomes using the same GAIS.

**Table 1:** Global Aesthetic Improvement Scale

Rating	Description
3	Very much improved in appearance
2	Much improved in appearance
1	Improved in appearance
0	No change in appearance
-1	Appearance worsened after treatment

Additionally, the Allergan Transverse Neck Lines (ATNL) score, a specialised tool for grading the severity of horizontal neck lines, was employed to objectively measure the degree of ageing-related changes in the patients' necks (Table 2). The authors initially assessed and then re-graded the patients using this score three months after treatment. A successful treatment outcome was defined as a minimum one-point improvement on both the GAIS and ATNL scales, indicating a noticeable and satisfactory enhancement in neck appearance.

**Table 2:** Allergan Tranverse Neck Lines Scale

Grade	Term	Descriptor
0	None	No transverse neck lines
1	Minimal	Superficial transverse neck lines
2	Moderate	Moderate transverse neck lines
3	Severe	Deep transverse neck lines
4	Extreme	Transverse neck furrows with excess skin

Results

Patient 1

**Figure 1:**
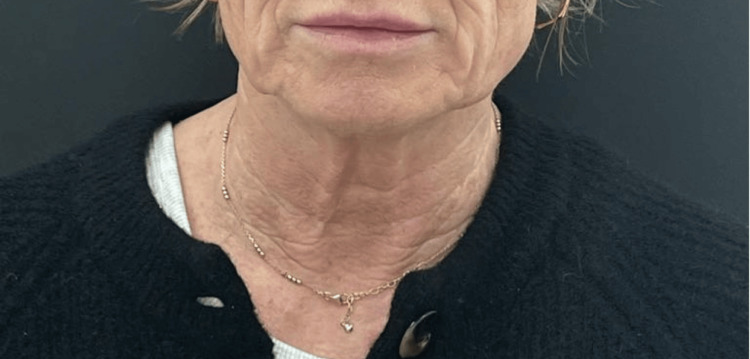
Patient 1, pre-treatment

**Figure 2:**
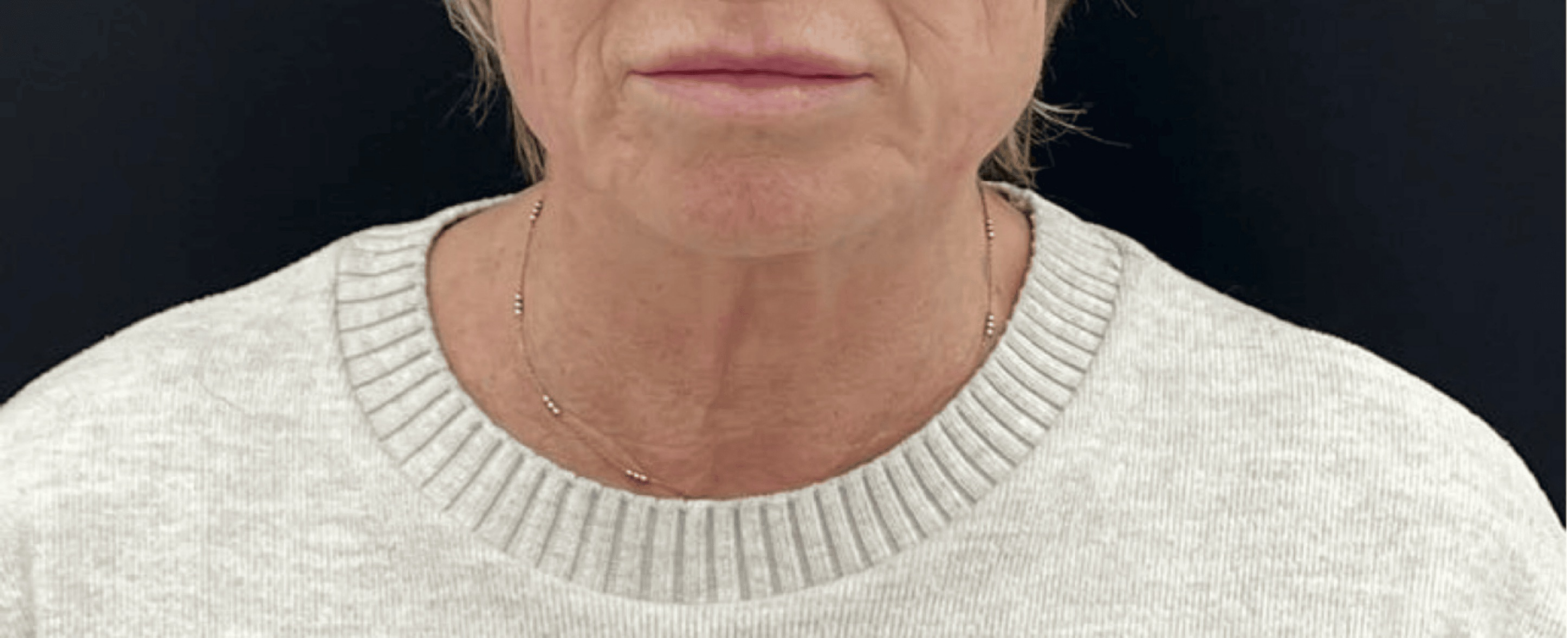
Patient 1, three months post-treatment

This 50-year-old woman presented with early-onset changes of ageing in the neck. She noticed an increase in the number of skin lines in the neck, as well as a thinning of the skin. She underwent treatment with diluted HArmonyCa™ as described. Her baseline ATNL score was 2, and post-treatment, this dropped to 1. Her GAIS score, according to the author and the patient, at three months post-treatment was 1, indicating an improvement in her appearance (Figures 1, 2).

Patient 2

**Figure 3:**
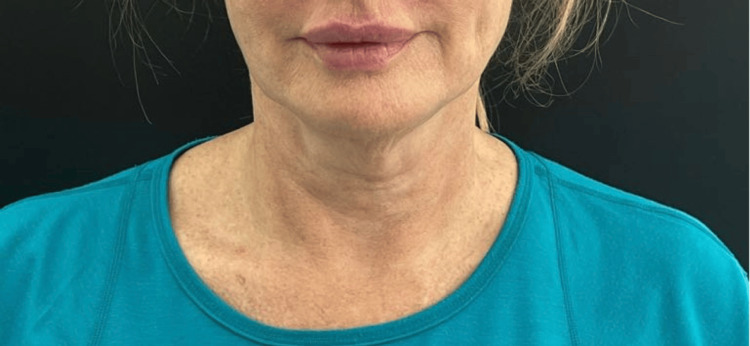
Patient 2, pre-treatment

**Figure 4:**
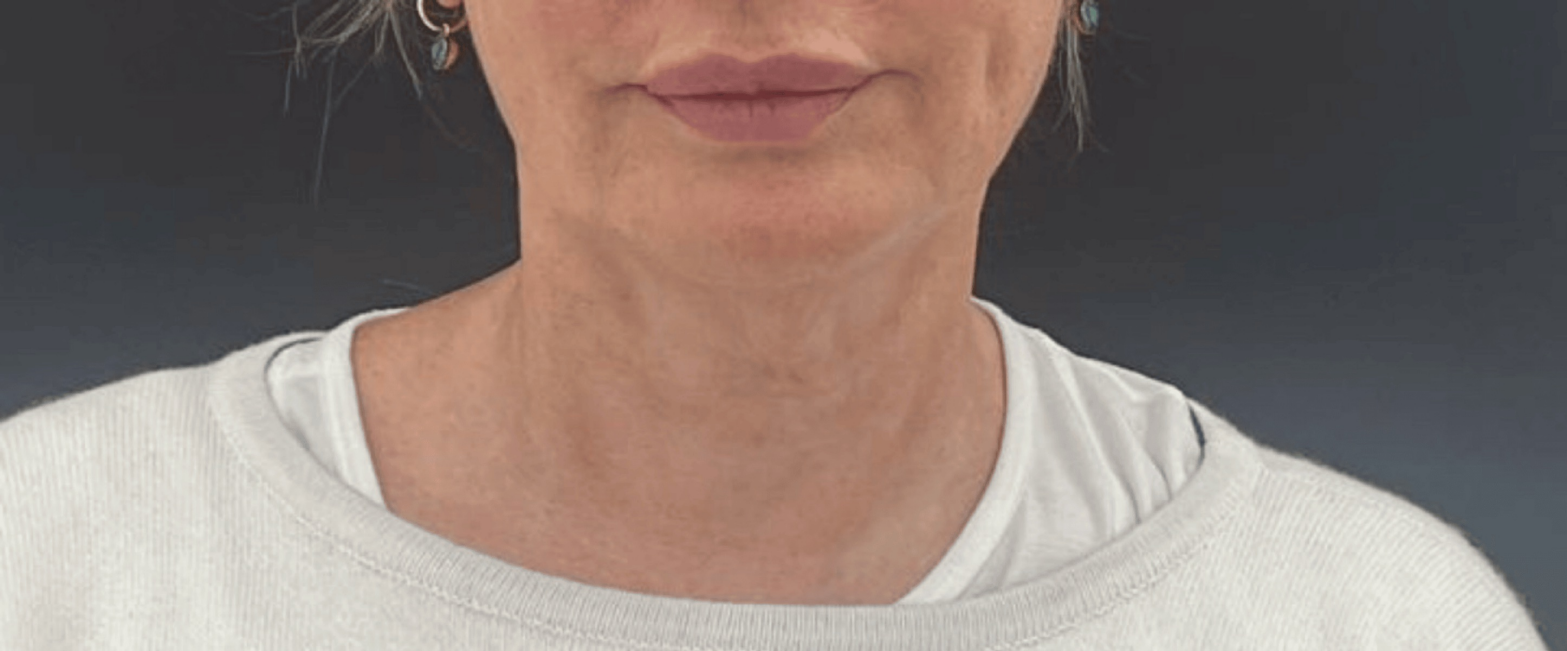
Patient 2, three months post-treatment

This was a 60-year-old woman with more advanced signs of ageing in her neck. Her baseline ATNL score was 4, and post-treatment, this dropped to 1. Her GAIS score at three months post-treatment, according to the author and the patient, was 2 (Figures 3, 4).

## Discussion

This case report represents the first documented use of diluted HArmonyCa™ for neck rejuvenation, demonstrating both safety and efficacy in two patients. The absence of adverse events, particularly nodules, granulomas, or vascular complications, aligns with prior studies of HArmonyCa in facial applications [3]. This is attributable to three key technical modifications. Firstly, the product was diluted 1:1 with sterile saline, which may have helped mitigate the development of lumps in the skin. It may also have helped reduce product cohesiveness. The usage of the cannula to minimise trauma, followed up by post-treatment massage, helped ensure even dispersion of the product and further prevent the development of lumps.

The observed improvement in the ATNLs and the GAIS three months post-treatment supports the collagen-stimulating effects of the HArmonyCa [3]. Limitations of the study were the small sample size of two patients and the short follow-up duration of three months, which precludes assessment of long-term efficacy.

## Conclusions

This pilot study demonstrates that diluted HArmonyCa™ is a safe and effective option for neck rejuvenation. The treatment offers a novel approach to addressing transverse neck lines, a historically challenging area for nonsurgical treatments. The absence of adverse events highlights the importance of technical adaptations such as 1:1 dilution, cannula delivery, and post-treatment massage, which optimise product integration in thin neck skin. Larger, controlled studies accompanied by long-term follow-up (greater than 12 months) would help assess the durability of the treatment. Objective measures, such as ultrasound, to quantify skin thickness could also be useful. This study lays the groundwork for HArmonyCa™ as a versatile tool in neck rejuvenation, bridging a gap in evidence-based aesthetic medicine.
